# Extraction of Artificial Teeth from the Gullet

**Published:** 1846-12

**Authors:** F. A. Bulley


### ARTICLE II.

### *Case of Extraction of Artificial Teeth from the Gullet.* By F. A. Bulley, Esq.

In the middle of the night of Saturday, February 7th, I was requested to attend, Mr. J. L., a tradesman, residing in Reading, who I understood had accidentally swallowed something that had become impacted in the œsophagus, the exact nature of which, however, from the patient's being unable to articulate clearly, I could not at first make out. From the fear and confusion produced by his distressing condition, as well as from the pressure of the foreign substance, whatever it might be, upon the trachea, his power of utterance was almost entirely gone, and he could merely mutter the words "plate," "teeth," "bone;" but beyond this he could give me no idea of his sufferings, but it was evident that unless I were fortunate enough to relieve him quickly, he would soon sink from suffocation.

As every moment seemed to be of consequence, I placed the patient in a chair without delay, and passing my left fore-finger down the œsophagus, as far as it would reach, I succeeded in just touching some hard substance, which appeared to be slowly making its way downwards; for, whether from my having accidentally assisted its passage by the slight pressure of my finger, or from some other cause, I could not after a few moments continue to feel it. Under these circumstance, I scarcely knew what was best to be done; the embarrassment of his breathing was momentarily increasing, and he seemed to be otherwise in a very critical condition.

The continued presence of the finger, however, in the gullet, producing a violent and excessive inverted action in the tube, and again, fortunately, brought the substance within its reach, but whether it was actually moved upwards, or whether, being still impacted, it was merely lifted up by the general spasmodic elevation of the œsophagus, which happens in the act of vomiting, I am not prepared to say.

I now passed a pair of short-curved polypus forceps by the side of the finger, and while the substance was still in contact with the end of the finger, succeeded in catching it between the blades. Feeling certain, by the touch, that it was of a metallic nature, and from the circumstance of his having, although indistinctly, spoken of his teeth, I concluded that he had swallowed a part of a set of artificial teeth, which he had been in the habit of wearing, but had usually removed at night. Continuing my finger in the gullet, I succeeded, by its assistance, in so easing the substance off the sides of the passage as to enable me to draw it upward, with some little difficulty, for the space of about an inch. When it had arrived opposite the central portion of the thyroid cartilage, its further progress was stopped, probably from some part of it having become entangled in the soft posterior wall of the larynx; the difficulty of breathing increased, and he seemed momentarily in danger of suffocation. I could now no longer afford any assistance by my finger, and as, moreover, its continued presence in the tube appeared to increase his suffocating feelings, I was obliged to withdraw it.

As there was, apparently, at this critical moment, no probability of extracting the substance, but by sheer force, and feeling unwilling, after getting it so far, to relinquish my hold of it, I endeavored, with the forceps in both hands, using a twisting motion to bring it into the pharynx, in which I fortunately succeeded, when the instrument slipped, and I ultimately removed the foreign body with my finger. The operation was followed by hæmorrhage to the extent of about four ounces, which was, however, subdued by the patient's drinking plentifully of cold water.

The substance extracted proved to be a gold frame-work or plate, such as is used for the fixture of artificial teeth, and measured exactly two and a half inches in its longest diameter, and one and a half inches in the shortest. Attached to its anterior edge were two artificial teeth, intended to supply the place of the two central incisors of the upper jaw, and on its under surface farther back, were two gold pins, which had been used to fix two other teeth, but which had become separated from the pins and lost some time before the accident. The hooked extremities of the plate were intended to fix it to two of the natural molar teeth, and it was to the circumstance of these teeth having become decayed, that the patient attributed the loss of its attachment to the jaw.

When he had somewhat recovered his power of speech and self-possession, he told me that he was awoke in the night by an odd sensation in his throat, as if, (to use his own expression,) "a part of the gristle of his windpipe had got out of order," the idea of his having swallowed the teeth not having crossed his mind; that he then passed his finger down his throat, and succeeded in pressing the body downwards, when he felt satisfied that he had "righted the part," and experienced little further inconvenience for nearly half an hour, during which time he thought he must have gone to sleep, as he felt suddenly to awake with a suffocating sensation, which continued increasing until the period when he reached my house.

His recovery was rapid and uninterrupted. The morning following the extraction, he complained of a fulness in the throat, and a great stiffness in the part, especially in swallowing. These symptoms were relieved by the application of ten leeches, followed by a bran poultice made with a decoction of chamomile and poppy heads. He was ordered to take some demulcent medicine, and to abstain entirely from solid food, his principal aliment consisting of very thin arrow-root, which he said relieved the stiffness and pain more than anything else he took. He had scarcely any fever during the period of his convalescence.

At the end of a week he was so far recovered as to be able to take toast and a little animal food, without inconvenience; from this time he gradually improved, losing all uneasiness in the part, and has remained perfectly free from any uneasiness in the throat ever since.

*Remarks.*—The foregoing case presents several points of interest, both in a surgical and physiological point of view. The little inconvenience the patient experienced at first in the passage of so large a substance down the œsophagus, if we may credit his own account, is a circumstance not easily explained. Possibly, not imagining the real cause of his sensations, and being in a half sleepy state, he might have thought that an accident to the windpipe had actually occurred, and he might have fancied he had righted it. How he could have remained so long afterwards, however, without feeling inconvenience, I am at a loss to say. The substance must have passed quickly and with comparative ease through that portion of the gullet which lies behind the larynx, aided by the pressure of his finger and the action of the constrictors of the pharynx, otherwise the sensation of suffocation would have earlier roused him to a sense of his danger; afterwards, when it had become impacted in that part of the œsophagus, where it was found, just below the level of the cricoid cartilage, it might not have pressed upon the trachea at first, until the natural propulsive action had changed its position in the tube. Again, if it were not of itself a cause of mechanical pressure upon the air tube, the disturbance which its continued presence in such a situation would produce to the nerves of the part, might sympathetically affect those of the trachea, and thus account for the suffocating feelings which after a time he experienced. In respect to the extraction I have very little to remark; had I been aware of the ragged and uneven nature of the substance to be extracted, I might perhaps have hesitated about using the degree of force sufficient to dislodge and remove it, for fear of lacerating the œsophagus and injuring some important vessel, which might have proved immediately fatal to the patient. On the other hand, finding it was after a time not drawn readily upwards, I might, acting under the same ignorance of its nature, have attempted to force it downwards with a probang—an operation which, even had I been able to accomplish its removal into the stomach, must have inevitably proved fatal to the patient. The most singular part of the case was the remarkably small amount of injury done to the œsophagus by the somewhat violent extraction, freedom from after bad consequences which the patient experienced, and the trifling hæmorrhage that followed it.

In conclusion, I trust that the publication of this case will not create any unnecessary alarm in the minds of those who are compelled to resort to mechanical contrivances as substitutes for the natural teeth, which, generally speaking, through the ingenuity of modern science, are admirably adapted to the purposes they are intended to answer.

The extreme rareness of an accident of the kind detailed, proves how little danger is attached to wearing them, provided ordinary attention is used to observe when they are getting out of repair. And I would only suggest, by way of caution to those who use them, that as soon as the fastenings appear to have become in the least insecure, or there is reason to believe that the exact relation of the artificial mechanism to the natural gum has become disturbed, as in course of time it must, the patient should discontinue wearing it at night, until it has undergone a thorough readjustment. Had the patient in this case given directions for the bands or fastenings of the plate to be extended to other and sounder teeth, when he found it was becoming loose from the loss of its old supports, the accident which nearly cost him his life would most probably never have happened.

